# How the Stomach Affects the Throat

**Published:** 1884-01

**Authors:** 


*How can the Stomach make the Throat Sore?*

A stroke against the elbow is felt at the fingers' end. When your foot is asleep, from sitting on a hard edge of wood for some time, the cause is at the point of pressure, and yet it tingles in the toes a yard off. A good knock on the head "makes the fire fly" at the eyes.

The condition of the throat is affected by the condition of the stomach, because a certain nerve branches off, one part of that nerve goes to the stomach, the other fork goes to the throat. The nerves are like the telegraphic wires, touch them at one end, and an effect is produced at the other. So if the nerves which supply the stomach are disordered, those in the throat are liable to become so too. Most of us have heard of "*heartburn*," some have felt it; it is a burning sensation, sometimes felt at the point familiarly called *the pit of the stomach*, and sometimes in persons who use their voice much, this same burning is felt at the little hollow at the bottom of the throat and the region of *Adam's apple*, and that is the spot where throat-ail is located.

I wish here to arrest the attention of clergymen, singers, teachers, and public speakers to this interesting inquiry.

If sour stomach, or *dyspepsia*, as physicians term it, causes burning or other sensations in the throat of clergymen and other persons who use their voice much, why does not sour stomach affect the throats of all, as the same nerve supplies branches to both throat and stomach? This is the reason: a slight stomach derangement does not affect the throat perceptibly, if the voice organs are in a strong, active, healthful condition, because they have vigor to repel disease. It is a law of the human frame, that an ailment is apt to make itself felt next, or most decidedly in that particular part of the body which at the time is weakest in the performance of its functions, and as the voice organs are at length made permanently feeble by these repeated uses and indiscretions, so being the next weakest part, disease flies there; thus it is too, that when such persons take cold, the throat being the weak part, feels it promptly.

A proper use of the voice strengthens the throat, and gives it a capability of resisting disease, just as a judicious use of any other muscles of the body increase their strength and health. But improper use, as just stated, by weakening, renders them more susceptible of disease of any kind, and specially of the stomach, in consequence of the nervous connection before described.

An injury done to any part of the body may be resisted, or if not, may be repaired by the curative energies of nature; but if these injuries are frequently repeated, the strength of nature is exhausted in endeavoring to make repairs, then she remains prostrate and powerless, and disease has unbridled sway.

When in any given case, a man is in a condition to have his throat affected by the state of his stomach, violence is offered the throat at each meal, three times a day, in time these effects last longer, until the effect of one meal reaches to another, and the throat is more or less ailing all the time.

But to follow up the case, how is it that persons have *sour stomach* or *heartburn?*

All understand that what is sweet cider to-day, is sour tomorrow; we look at it and find it in constant motion, it is "*working*," *fermenting*. When food is taken into a healthy and well acting stomach, it is in a short time *digested*, that is, converted into a kind of liquid, no lumps or any thing of the sort in it, just as when you place a great many bits of ice and snow in a glass of water, the mass soon becomes all fluid alike. The food is made into this one fluid substance by the action of the stomach and what pertains to it. But the amount of food which the stomach can thus turn into a liquid form, is limited, just as if you put a certain amount of ice lumps in a glass of water, that water will melt them, but if you put in too many, none of them are wholly melted, and it remains a mixture of water, spears of ice, and solid ice. When then, more food is taken into the stomach at any one time than it can convert into a homogenous fluid, it remains in lumps more or less, and it is said to be *undigested*, and begins immediately to ferment, to become sour and produces in the stomach the same sensation that swallowing vinegar causes in the throat, a burning.

We see then, that sour stomach is caused by eating more than the stomach can digest. But how are we to tell how much the stomach can digest? In the same manner precisely as each one may ascertain to a quarter of an hour how much sleep he needs

Observe nature. The brutes are regulated in all these things by instinct, to us the nobler reason is given, and it must be our guide. We must observe and judge.

What one man eats or drinks in quality or quantity is no guide for any other man, any more than the amount of labor one can perform, is the criterion for another. Each man must for himself bring his own observation and judgment to bear on the question, *How much must I eat?* The general rule is, Do not eat so much, as to cause any unpleasant sensation after wards.

If you at any time take a meal, and afterwards within an hour or two feel uncomfortably, then what you have eaten, *does not agree with you*; you have eaten, either in quantity or quality what your stomach cannot digest. Nine times out of ten, it is the quantity and not the quality, which does the mischief.

When persons have been ailing some time, almost every thing they eat or drink, sours on the stomach, even a cup of tea or a glass of cold water, or toasted bread, gives sourness, or weight, or oppression, or some other ill feeling; in time, the throat begins to feel tired, dry, or to burn, or smart, or is clogged up a little and we are all the time clearing it away; this is "*Dyspeptic Throat-Ail*," or Clergymen's Sore Throat.

Throat-ail then being generally located in the stomach—what is the use of gargling the throat with acids and metallic preparations, which destroy the teeth? and what is the use of swabbing out the throat with nitrate of silver, when the source [illegible]

relief, but it is not permanent, it cannot be, for it is merely covering a black spot on the wall with whitewash; the spot is not seen, but it is there still; but unlike the black spot, which is statu quo, the disease, though covered, is burrowing and spreading still.

COLD FEET

Often produce a burning sensation in the throat, which if allowed to continue in operation, ultimately undermines the health; the reason is, less blood being in the feet than is natural, there is an extra amount at the other end of the body; can any thing be more absurd than to clip off a man's palate, whack out his tonsils, and "burn out his throat," for such an ailment; can that send warmth to the feet? can we purify the fountain, by purifying the stream? When will men learn to think for themselves?

My experience is, *Throat-Ail is not to be radically and permanently cured in any case, except by rectifying first, and then building up the general health of the system*, and that requires time, determination, and systematic habits of rational life.

### SHAVING THE BEARD.

It appears by experiment that the beard, if shaved, grows four to five times faster than if unshorn. In this calculation, an item is omitted which it is difficult to estimate, *i. e.*, the stimulus given the beard, by the first application of the razor in adolescence, the experiments being made upon beards after they have acquired an unnaturally rapid growth. The effect of this early stimulus may be fairly counted at double the natural growth; then reckoning the difference in size and weight of the fibre, which is treble, and we find the frightful truth to be, that we raise thirty times the natural quantity of beard! Thus it is evident that the true beard is exhausted at a very early age, after which the system is forced to supply a substitute. Now nature will not submit with impunity to extraordinary demands upon her vigor, and that which requires her to produce in a life time thirty times as much beard as she was first inclined to, must certainly be considered as such. She is fatigued in proportion to the effort, let the particular kind be what it may; although her recuperative powers are great, she insists upon having repose, even when working at a rate chosen by herself. If that repose is denied her, she takes her revenge by breaking down the mechanism.

### COUGHING

A gentleman called upon us recently, who actually escaped from the fangs of consumption some years ago; and we are induced to present the circumstances.

"You speak of coughing continually. Let me suggest to you the query, whether this is not unnecessary and injurious? I have long been satisfied, from experience and observation, that much of the coughing, which precedes and attends consumption, is voluntary. Several years ago, I boarded with a man who was in the incipient stages of consumption. I slept in a chamber over his bed-room, and was obliged to hear him cough continually and distressingly. I endured the annoyance, night after night, till it led me to reflect whether something could not be done to stop it. I watched the sound which the man made, and observed that he evidently made a voluntary effort to cough. After this I made several experiments on myself, and found that I could prevent myself from coughing, sneezing, gaping, &c., in case of the strongest propensity to these acts, by a strenuous effort of the will. Then I reflected that coughing must be very irritating and injurious to the delicate organs that are concerned in it, especially when they are in a diseased state. What can be worse for ulcered bronchia, or lungs, than the violent wrenching of a cough? It must be worse than speaking. A sore on any part of the body, if it is constantly kept open by violent usage, or made raw again by a contusion just when it is healing (and of course begins to itch) will grow worse, and end in death. Certainly, then, a sore on the lungs may be expected to terminate fatally, if it is constantly irritated, and never suffered to heal; and this, it seems to me, is just what coughing does for it. On the strength of such considerations as these, I made bold to ask the man if he could not stop coughing. He answered, no. I told him what I thought about it, as above. He agreed to make a trial; and on doing so, he found to his surprise that he could suppress his cough almost entirely. The power of the will over it increased as he exercised it, and in a few days he was mostly rid of the disposition to cough. His health, at the same time, evidently improved, and when I last saw him, he was in strong hopes of getting out of death's hands."

**Parental Responsibility.**—The father molds the head; the mother, the heart; the father appeals to the understanding; the mother, to the affections; the father prepares for time; the mother, for eternity. Happy the children who heed the wise teachings of both.

